# Molecular Characterization of Closely Related H6N2 Avian Influenza Viruses Isolated from Turkey, Egypt, and Uganda

**DOI:** 10.3390/v13040607

**Published:** 2021-04-02

**Authors:** Yavuz Mercan, Gladys Atim, Ahmed E. Kayed, M. Ekin Azbazdar, Ahmed Kandeil, Mohamed A. Ali, Adam Rubrum, Pamela McKenzie, Richard J. Webby, Bernard Erima, Fred Wabwire-Mangen, Qouilazoni A. Ukuli, Titus Tugume, Denis K. Byarugaba, Ghazi Kayali, Mariette F. Ducatez, Zeynep A. Koçer

**Affiliations:** 1 Emerging Viral Diseases Laboratory, Izmir Biomedicine and Genome Center, 35340 Izmir, Turkey; yavuz.mercan@msfr.ibg.edu.tr (Y.M.); ekin.azbazdar@msfr.ibg.edu.tr (M.E.A.); 2 Izmir International Biomedicine and Genome Institute, Dokuz Eylul University, 35340 Izmir, Turkey; 3 Makerere University Walter Reed Project, P.O. Box 7062 Kampala, Uganda; gatim@muwrp.org (G.A.); berima@muwrp.org (B.E.); fwabwire@musph.ac.ug (F.W.-M.); qukuli@muwrp.org (Q.A.U.); ttugume@muwrp.org (T.T.); dkb@covab.mak.ac.ug (D.K.B.); 4 Center of Scientific Excellence for Influenza Viruses, National Research Centre, Giza 12311, Egypt; Ahmed.Elsayed@human-link.org (A.E.K.); Ahmed.Kandeil@human-link.org (A.K.); mohamedahmedali2004@yahoo.com (M.A.A.); 5 St Jude Children’s Research Hospital, Memphis, TN 38105, USA; Adam.Rubrum@stjude.org (A.R.); Pamela.McKenzie@stjude.org (P.M.); richard.webby@stjude.org (R.J.W.); 6 School of Public Health, Makerere University, P.O. Box 7062 Kampala, Uganda; 7 College of Veterinary Medicine, Makerere University, P.O. Box 7062 Kampala, Uganda; 8 Department of Epidemiology, Human Genetics, and Environmental Sciences, University of Texas, Houston, TX 77030, USA; ghazi@human-link.org; 9 Human Link, Dubai, United Arab Emirates; 10 IHAP, UMR1225, Université de Toulouse, INRAe, ENVT, 31076 Toulouse, France; mariette.ducatez@envt.fr

**Keywords:** avian influenza virus, waterfowl, migratory birds, molecular markers, reassortment

## Abstract

Genetic analysis of circulating avian influenza viruses (AIVs) in wild birds at different geographical regions during the same period could improve our knowledge about virus transmission dynamics in natural hosts, virus evolution as well as zoonotic potential. Here, we report the genetic and molecular characterization of H6N2 influenza viruses isolated from migratory birds in Turkey, Egypt, and Uganda during 2017–2018. The Egyptian and Turkish isolates were genetically closer to each other than they were to the virus isolated from Uganda. Our results also suggest that multiple reassortment events were involved in the genesis of the isolated viruses. All viruses contained molecular markers previously associated with increased replication and/or pathogenicity in mammals. The results of this study indicate that H6N2 viruses carried by migratory birds on the West Asian/East African and Mediterranean/Black Sea flyways have the potential to transmit to mammals including humans. Additionally, adaptation markers in these viruses indicate the potential risk for poultry, which also increases the possibility of human exposure to these viruses.

## 1. Introduction

Avian influenza viruses (AIVs) are a major public and animal health concern due to infection in poultry and documented zoonotic potential. Although there is an increasing awareness towards all AIVs, most studies focus on H5 and H7 subtypes as some strains are highly pathogenic and human infections with these viruses are reported more frequently than with other AIVs. However, low pathogenic avian influenza (LPAI) viruses also deserve attention since they contributed to the emergence of four pandemics [1,2,3,4] and have the potential to cause other pandemics in the future. Since the RNA dependent RNA polymerase (RdRp) of influenza viruses lacks proofreading mechanisms, many mutations occur in the viral genome that may provide AIVs an opportunity for adaptation to a new host. H6 influenza viruses are one of the most prevalent AIV subtypes in migratory birds and poultry and have been detected in humans [5,6,7,8]. After their first detection in turkeys in 1965, H6 viruses have been detected frequently in wild birds and poultry [5,6,7,8]. Recently, H6N2 viruses have been detected in poultry and wild aquatic birds in India and Egypt, respectively [7,8]. Genetic characterization of the viruses isolated in India indicated that these viruses were reassortants of different viruses of Eurasian origin [7]. The H6N2 virus that was isolated from wild birds in Egypt contained molecular markers related to increased pathogenicity in mammals (in PB2, PB1-F2, M1, NS1), as well as amantadine resistance (in M2) [8].

Avian origin H6 viruses have been shown to be able to infect and even cause mortality in mice without prior adaptation [9,10]. Avian H6N2 viruses were also shown to be capable of direct transmission between guinea pigs, a model for human transmission [11]. These studies are consistent with the evidence for H6 infections in humans. In a serological study conducted in the US, veterinarians working in close contact with birds were found to be seropositive for H6 viruses [12]. In 2013, an H6N1 virus was isolated from a hospitalized 20-year-old woman in Taiwan [13]. Genetic analyses indicated that the human virus originated from chickens and contained markers that provide amantadine resistance (N31 in M2) and increased affinity for mammalian type sialic acid (SA) receptor binding (S228 in HA) [13]. Another study showed that 0.4% of 15,000 people participating in poultry-related work were found to be seropositive for H6 viruses [14]. Considering that they are frequently detected in wild birds and poultry, and sporadically in humans, H6 AIVs have a potential to cause outbreaks affecting public and/or animal health.

In this study, we sequenced the whole genomes of four H6N2 viruses isolated during ongoing surveillance studies in 2017–2018 in Turkey (one), Egypt (two), and Uganda (one) which are located on the Mediterranean/Black Sea and West Asian/East African Flyways. These flyways are connected to each other through a branch of Mediterranean/Black Sea Flyway which passes through Turkey and continues over Syria and Egypt to South Africa [15]. On the other hand, West Asian/East African Flyway passes through the eastern part of Turkey and continues over Egypt to South Africa through Uganda. Thus, Egypt plays an important role for possible reassortment events between AIVs from migratory birds on these two flyways. Phylogenetic analyses were performed to determine the origins of each gene segment and to investigate the genetic relatedness of these four H6N2 influenza viruses, as well as possible reassortment events. Additionally, the whole genomes of these viruses were examined to identify molecular markers considered to enhance zoonotic risk.

## 2. Materials and Methods

### 2.1. Legal Permits

Field studies in Turkey were approved by the scientific research permit (No: 72784983-488.04-174660, Date: 15 August 2017) granted by the Republic of Turkey Ministry of Agriculture and Forestry. The isolation of H6N2 virus in Turkey was performed under the scientific research permit (No: 71037622-325.01-E.3135467, Date: 14 October 2019) granted by the Republic of Turkey Ministry of Agriculture and Forestry. A special permit was received from the Republic of Turkey Ministry of Agriculture and Forestry to publish the data on the molecular and phylogenetic characterization of H6N2 isolate from Turkey (No: 71037622-325.01-E.3784816, Date: 9 December 2019). Sampling wild birds in Egypt was approved by the Ethical Committee at the National Research Centre, Giza, Egypt (Registration No: 16034). Sampling in Uganda was carried out with the approval from the Research Committee of the School of Biosecurity, Biotechnology and Laboratory Sciences (No: VET-0804) and Uganda National Council for Science and Technology (No: HS-426).

### 2.2. Viruses

Virus isolation was performed by inoculating fecal, oropharyngeal, or cloacal samples into allantoic cavities of 10-days old embryonated chicken eggs and incubated at 35 °C for 3 days as described previously [10].

The H6N2 viruses analyzed in this study were obtained from the ongoing surveillance studies in Uganda, Egypt, and Turkey in 2017–2018. A/aquatic bird/Gediz Delta/1/2018 (H6N2) (GD/18) virus used in this study was isolated from a fecal sample of a wild aquatic bird that was collected in March 2018 in Gediz Delta/Izmir Bird Paradise, Izmir, Turkey. The GD/18 was the only virus isolate obtained from 156 AIV positive samples by PCR among 500 fecal samples that were collected from the same region during 2017–2018. A/teal/Egypt/MBD1566C/2018 (H6N2) and A/teal/Egypt/MBD1556OP/2018 (H6N2) (EG/1566C and EG/1556OP, respectively) were isolated in Egypt from cloacal and oropharyngeal samples obtained from healthy trapped teals in a live bird market located along the Egyptian Eastern Mediterranean coast in January 2018 and were among 17 positive samples out of 740 collected swabs during the 2017–2018 season. A/wild bird/Uganda/MUWRP-731/2017 (H6N2) (UG/731) was isolated from a fecal sample collected in 2017 on Lake Victoria in Uganda. The UG/731 was the only virus isolate obtained from 42 AIV positive samples by PCR with high Ct values among 574 fecal samples of waterfowls collected in 2017–2018 in Uganda.

### 2.3. Whole Genome Sequencing

Viral RNA from infected allantoic fluid was extracted using a QIAamp Viral RNA Mini Kit (Qiagen, Hilden, Germany) according to the manufacturer’s instructions. Each gene segment of the virus was amplified using One Step RT-PCR Kit (Qiagen, Hilden, Germany) with universal primers for each segment [16]. Amplicons were purified using a QIAquick Gel extraction kit (Qiagen, Hilden, Germany) following the manufacturer’s instructions. Purified amplicons were sequenced by Sanger (Turkish isolate) or by Illumina next generation sequencing (Egyptian and Ugandan isolates) as previously described [17].

### 2.4. Nucleic Acid and Amino Acid Identities of Virus Isolates

Nucleotide and amino acid pairwise distances between each gene of the four H6N2 isolates were calculated in MEGA X software [18] using the maximum composite likelihood approach.

### 2.5. Sequence Analyses

Sequence reads of each segment were assembled using SeqMan Pro software of DNASTAR Lasergene 16 (DNASTAR, Madison, WI, USA). Assembled contigs were aligned using BioEdit software v7.2.5 [19] and each amino acid position was examined to detect molecular markers previously identified as having phenotypic impact.

### 2.6. Phylogenetic Tree Construction

Phylogenetic trees were drawn using the Maximum Likelihood method based on the Hasegawa-Kishino-Yano model [20] with 1000 bootstraps on MEGA6 [21]. When genetic diversity among Turkish, Egyptian and Ugandan H6N2 viruses along with the limited number of full genome data for related viruses on public databases were considered, the viruses that closely clustered with either Turkish, Egyptian, or Ugandan gene segments in most phylogenetic trees were selected. Thus, A/domestic duck/Georgia/1/2016 (H4N6), A/Teal/Markeev/13-3-11/2016 (H5N2), A/teal/Egypt/MB-D-487OP/2016 (H7N3), and A/duck/Moscow/5662/2018 (H1N2) were selected as reference strains for our viruses.

### 2.7. Sequence Accession Numbers

The nucleotide sequences for each virus were deposited to NCBI GenBank and can be accessed by the following accession numbers: MT783429-to-MT783436 for GD/18; MT570358-to-MT570365 for EG/1566C; MT570350-to-MT570357 for EG/1556OP; MT703748-to-MT703755 for UG/731.

## 3. Results

### 3.1. Genetic Relatedness of H6N2 Isolates from Turkey, Egypt, and Uganda

To identify the genetic similarities of each gene of the H6N2 viruses isolated from Turkey, Egypt, and Uganda, pairwise distances were calculated. All four viruses showed moderate to high identity/homology at both nucleotide (91–100%) and amino acid levels (~96–100%) (Supplementary Tables S1 and S2). The most conserved gene among the four viruses was the M gene with pairwise nucleotide identities ranging between 97% and 100%. The M1 protein sequences of all four H6N2 isolates were identical. The most variable gene was PB2 (pairwise nucleotide identities 91% to 99%) (Supplementary Table S1) despite amino acid sequence identities of ~99%. The most variable protein was NA (pairwise amino acid identities 95% to 99%) (Supplementary Table S2). The UG/731 NS1 showed ~4% amino acid sequence divergence from that of the other three viruses, while the NEP of the UG/731 was highly similar (98–99%) to that of other three isolates (Supplementary Table S2). Overall, EG/1566C and EG/1556OP were genetically very similar to each other with GD/18 being more similar to these viruses than to UG/731. It is noteworthy that the polymerase genes of EG/1556OP were more similar to GD/18 than to EG/1566C (Supplementary Tables S1 and S2).

### 3.2. Amino Acid Markers of Pathogenicity, Mammalian Adaptation and Antiviral Resistance

**Figure 1:**
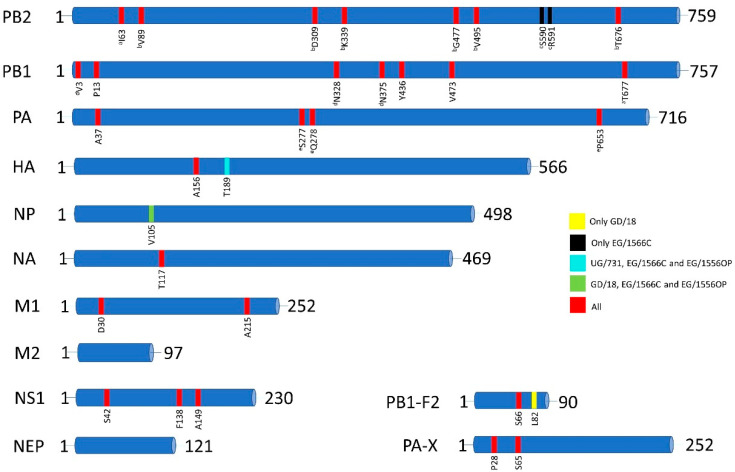
Molecular markers of mammalian adaptation, pathogenicity and antiviral resistance identified in GD/18, EG/1566C, EG/1556OP, and UG/731. Markers identified only in GD/18 are labelled with yellow; only in EG/1566C are labelled with black; in UG/731, EG/1566C and EG/1556OP are labelled with turquoise; in GD/18, EG/1566C, EG/1556OP are labelled with green rectangles on respective proteins. Markers which are conserved in all viruses are labelled with red rectangles. Markers tagged with superscript letters were previously reported to be present together for their corresponding functional outcomes. The numbers on each gene denote the amino acid numbers.

To identify molecular markers in the H6N2 viruses associated with pathogenicity, mammalian adaptation and/or antiviral resistance, the whole genome of each virus was examined. All four viruses had such markers that had been previously described in the literature [22,23,24]. In total, 29 to 31 amino acid markers were detected in the genomes of the H6N2 isolates. These molecular markers were detected in 10 of 12 proteins of H6N2 viruses, with the exceptions of M2 or NEP proteins (Figure 1, Table 1). A detailed list of the markers and their respective potential functional outcomes are given in Supplementary Table S3.

The PB2 of all four viruses possessed avian signatures at positions 627 and 701. PB2 of GD/18, UG731 and EG/1556OP viruses harbored seven amino acid markers that are correlated with increased polymerase activity and virulence in mice/mammals while EG/1566C virus harbored eight markers (Table 1 and Supplementary Table S3). Substitutions V89, D309, K339, G477, V495, and T676 were shared among all viruses (Figure 1). These substitutions were shown to be related to enhanced polymerase activity and increased virulence of an H5N1 virus in mice [25]. Additionally, EG/1566C contained S590 and R591 which confer mammalian adaptation of 2009 pandemic H1N1 virus [26]. All viruses also contained I63 in PB2 which was shown to increase the virulence of H5N1 virus in mice together with the presence of T677 in PB1 that was also present in all four isolates [27].

We detected seven amino acid markers in PB1, all of which were common in all four viruses. Among these, four markers (V3, N328, N375 and Y436) were shown to be correlated with increased polymerase activity and/or increased virulence of H5N1 viruses in mammals [28,29] (Supplementary Table S3). Of these markers, Y436 was also shown to contribute to increased pathogenicity in mallards [29]. P13 was reported to be associated with adaptation of an avian H7N7 to mammalian species [30]. Coexistence of V3, N328 and N375 was found to be responsible for increased replication ability of A/Vietnam/1203/04 (H5N1) in mammals [28,31]. Another marker, V473, which was also conserved in the 2009 pandemic H1N1 virus, was correlated with increased polymerase activity and replication efficiency in mammalian cells [32]. A well-known marker, S66, was common in the PB1-F2 of all four H6N2 isolates and was shown to be correlated with increased virulence in mammals [33]. S66 was also found in the 1918 pandemic H1N1 viruses and correlated with increased pathogenicity [34,35]. The other marker, L82, found only in the PB1-F2 of GD/18, has been shown to promote secondary infection with Streptococcus pneumoniae in infections with 1968 pandemic strain A/Hong Kong/1/68 (H3N2) virus [36].

The PA of all viruses contained four amino acid markers. Of these, three markers (S277, Q278, P653) were shown to contribute to mammalian adaptation of an avian H10N8 virus [37]. In addition to these, all PAs contained A37 which was previously demonstrated to be correlated with elevated levels of replication and propagation of H7N9 viruses in mammalian cells [38]. The PA-X of all four viruses also harbored P28 and S65. Presence of these markers has been shown to be correlated with increased host transcriptional shutoff activity of pandemic A/California/04/2009 (H1N1) virus and the effect was maintained when these mutations were introduced into H3N2, H5N1 and H7N9 viruses [39].

The cleavage sites of the HA proteins of all four H6N2 viruses contained a single arginine (R/GLF) which is a determinant of low pathogenicity. We detected one marker, A156 (H5 numbering), in the HA of all viruses that was shown to alter the receptor affinity of H5N1 viruses towards α-2,6 sialic acid (SA) receptors [40,41]. The HA of UG/731, EG/1566C and EG/1556OP also harbored T189 which was shown to provide increased binding affinity for H5N1 viruses to α-2,6 SA receptors [42].

The NP proteins of GD/18, EG/1566C and EG/1556OP harbored V105 that was found to provide adaptation of highly pathogenic duck origin H5N1 viruses to chickens [43].

We detected one marker, T117 (N2 numbering), in the NA of all viruses which was shown to be associated with decreased susceptibility of H5N1 viruses to the antivirals oseltamivir and zanamivir [44]. 

In the M1 protein, two amino acid markers which are associated with increased virulence of H5N1 viruses in mammals were detected [45]. All four viruses possessed both D30 and A215 on their M1 proteins. No amino acid markers were detected in the M2 protein.

In NS1, three amino acid markers were detected (S42, F138 and A149) in all four H6N2 viruses. Of these markers, F138 was reported to increase the replication of an H5N1 virus in mammalian cells when it coexists with avian type PDZ-binding motif (ESEV) [46]. All four H6N2 viruses in this study possessed ESEV motif at their PDZ-binding site. The other marker S42 was shown to be associated with increased virulence of H5N1 viruses [47]. The third marker, A149, which was identified in avian H5N1 viruses, was indicated to be able to antagonize the induction of interferon protein levels in chicken embryo fibroblasts [48]. No amino acid markers were detected in NS2 protein.

### 3.3. Phylogenetic Relatedness of H6N2 Isolates from Turkey, Egypt, and Uganda

To determine the genetic origins of the viral genes and the reassortment events that resulted in the genesis of the isolated H6N2 viruses, phylogenetic analyses were performed. The genetic groups were mainly defined as per (i) the topologies of trees and (ii) the bootstrap values. No genetic distance threshold was used here as they differed for distinct gene segments. In addition to the sequences generated in the present study, reference sequences were included, either as they were geographically relevant, or as the first 50 NCBI Blast hits for each gene segment and each isolate.

**Figure 2:**
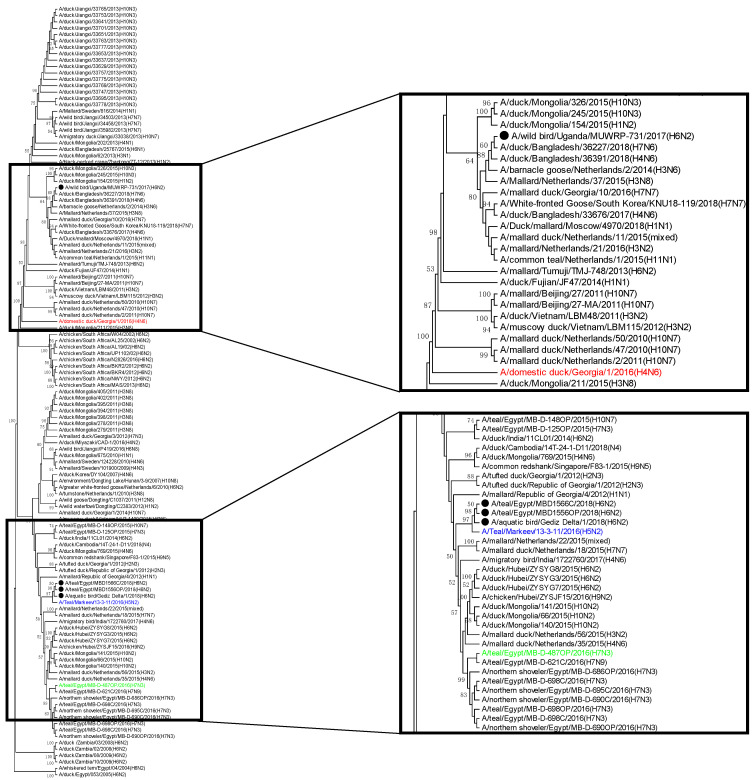
Phylogenetic relationships between the PB2 gene of GD/18, EG/1566C, EG/1556OP, and UG/731. Phylogenetic tree was constructed using the Maximum Likelihood method based on the Hasegawa-Kishino-Yano model. All four viruses in this study were indicated with black circles. According to the phylogenetic analysis, the PB2 of GD/18, EG/1566C and EG/1556OP clustered with A/teal/Markeev/13-3-11/2016 (H5N2) (blue). However, the PB2 of UG/731 was genetically closer to that of AIVs circulated in the Netherlands and Bangladesh. Reference viruses (most closely related strains for at least one gene segment) are color-coded: A/domestic duck/Georgia/1/2016 (H4N6) in red, A/Teal/Markeev/13-3-11/2016 (H5N2) in blue, and A/teal/Egypt/MB-D-487OP/2016 (H7N3) in green.

The PB2 genes of GD/18, EG/1566C and EG/1556OP clustered together with PB2 of A/Teal/Markeev/13-3-11/2016 (H5N2). The cluster also shared a common ancestor with PB2 sequences from viruses detected in Egypt in 2016. The PB2 of UG/731 instead clustered with PB2 sequences of the viruses from Bangladesh (2018) and the Netherlands (2014–2015) and shared a common ancestor with a virus isolated from a domestic duck in Georgia (Figure 2).

The PB1 genes of GD/18, EG/1566C and EG/1556OP formed a cluster with that of two viruses from Korea (2018) and Mongolia (2015). However, like the PB2 gene, UG/731 was genetically more distant than the other three isolates. The PB1 of UG/731 was genetically closer to that of AIVs that circulated in Egypt in 2016 and it shared a common ancestor with PB1 genes of AIVs from Mongolia isolated in 2018 (Supplementary Figure S1). The PA genes of GD/18, EG/1566C and EG/1556OP also clustered together forming a distinct group that shared the most recent common ancestor with the PA genes of AIVs from Bangladesh. This group was related to the AIVs from the Netherlands. On the other hand, the PA of UG/731 was found to be distant from those of the other isolates. The PA of UG/731 clustered with AIVs from Mongolia (2015), Egypt (2015–2016) and Bangladesh (2014–2015) (Supplementary Figure S2).

**Figure 3:**
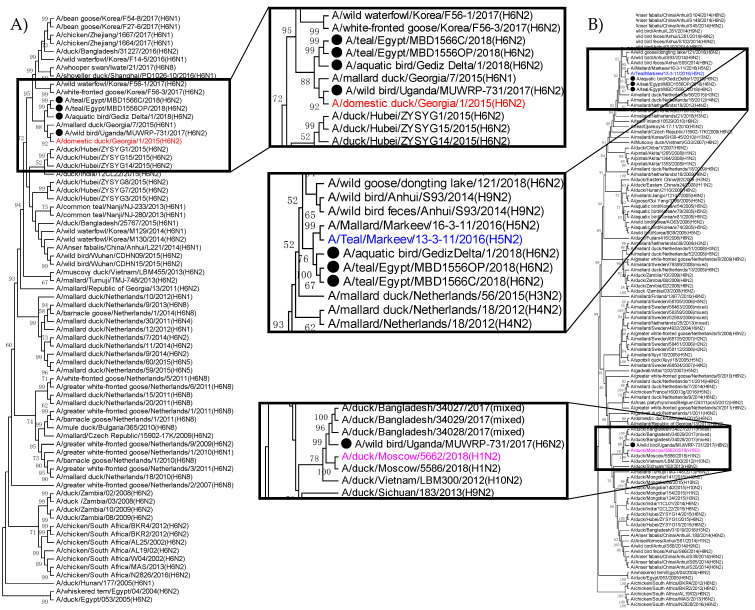
Phylogenetic relationships between GD/18, EG/1566C, EG/1556OP, and UG/731 based on (**A**) HA and (**B**) NA genes. All four viruses were indicated with black circles. Phylogenetic trees were constructed using the Maximum Likelihood method based on the Hasegawa-Kishino-Yano model. Phylogenetic analysis of HA gene showed that all four viruses were clustered together with A/domestic duck/Georgia/1/2015 (H6N2) strain (red). NA gene of GD/18, EG/1566C and EG/1556OP were clustered with A/Teal/Markeev/13-3-11/2016 (H5N2) (blue). UG/731 was closely related to N2 viruses from Bangladesh, Moscow and Vietnam including A/duck/Moscow/5662/2018 (H1N2) (purple).

The HA genes of GD/18, EG/1566C, EG/1556OP, and UG/731 clustered together and shared a common ancestor with H6 viruses from Georgia (Figure 3A). However, among all four H6N2 isolates, the HA of UG/731 was slightly distant from that of the other three.

The NP genes of EG/1566C and EG/1556OP clustered together forming a distinct group within a cluster dominated by sequences from Egypt (2016–2017). UG/731 was also located in the same cluster but rather distant from EG/1566C and EG/1556OP. The NP of GD/18 was found to be quite distant from EG/1566C, EG/1556OP, and UG/731 as it clustered with NP genes of AIVs from Europe (Supplementary Figure S3).

Like the polymerase genes and HA gene, the NA genes of GD/18, EG/1566C, and EG/1556OP were also genetically similar. They shared a common ancestor with H5N2 viruses detected in Ukraine in 2015 (Figure 3B). However, the NA of UG/731 was genetically closer to the N2 viruses from Bangladesh (2017), Moscow (2018) and Vietnam (2012). The tree topology indicates that UG/731 is distantly related to the other isolates (Figure 3B).

M gene sequences of EG/1566C and EG/1556OP were identical to each other and were similar to the M genes of AIVs from Mongolia that circulated in 2017. Although the M gene of GD/18 was genetically related to A/duck/Bangladesh/33676/2017 (H4N6), it shared a common ancestor with the Egyptian isolates, EG/1566C and EG/1556OP. As opposed to the Turkish and Egyptian isolates, the M gene of UG/731 was found to be closely related to that of European and African AIVs (Supplementary Figure S4).

The NS genes of EG/1566C and EG/1556OP isolates were also identical to each other and closely related to the AIVs circulated in Mongolia, as well as the Netherlands and Egypt. The NS genes of UG/731 and GD/18 were genetically closer to each other and clustered with the NS of A/duck/Bangladesh/36227/2018 (H7N6), although they shared a common ancestor with the AIVs in the Netherlands that circulated between 2006–2011 (Supplementary Figure S5).

Taken together, Table 2 summarizes the genetic constellation of our four isolates. It clearly highlights reassortment events on the flyways with HA, M, and NS gene segments of common origin for the four viruses. In addition, it clearly shows that the Turkish and Egyptian viruses are closely related except for NP.

## 4. Discussion

Mediterranean/Black Sea and West Asian/East African Flyways are two of the major migratory bird flyways covering most of Asia, Africa, and Europe. Turkey is located in the Mediterranean/Black Sea Flyway and contains several branches of the West Asian/East African Flyway. Egypt is located where the Mediterranean/Black Sea and West Asian/East African Flyways merge and Uganda is located in the West Asian/East African Flyway. Locations where two or more flyways intersect host high numbers of migratory birds from different origins; thus, can be hot spots for the emergence of novel AIVs with outbreak potential. Additionally, the three countries are connected to each other through the Mediterranean/Black Sea and West Asian/East African Flyways which pass through Turkey and continues over Syria and Egypt to South Africa [15]. Thus, surveillance studies carried out in these regions are important to evaluate the risk for the emergence of novel AIVs that have potential for zoonosis.

During the ongoing surveillance studies carried out in Turkey, Egypt, and Uganda, four avian influenza A viruses with H6N2 subtype were isolated. Overall, the viruses were genetically similar to each other at nucleotide and amino acid levels (Tables S1 and S2), with the Ugandan virus being the most diverse of the four. Some gene segments of the H6N2 isolates were similar to those of H5N2 (A/Teal/Markeev/13-3-11/2016) and/or H7N3 (A/teal/Egypt/MB-D-487OP/2016) viruses (Table 2). An H6N1 virus from a teal (A/teal/Hong Kong/W312/97 (H6N1)) showing high homology with the highly pathogenic zoonotic H5N1 virus in Hong Kong in 1997 (A/Hong Kong/156/97 (H5N1)) was previously reported [49]. In the same study [49] it was also shown that the internal genes of H6N1, H5N1, and H9N2 viruses were quite similar indicating possible reassortment events among those subtypes.

Although natural reservoirs of AIVs are wild aquatic birds, these viruses can cross species barriers and transmit to mammals including humans. H6 viruses are one of the most frequently detected viruses in wild birds and poultry. Moreover, experimental studies indicate that H6 viruses isolated from wild birds can infect mammals and cause mortality [9,10,50]. Identification of the first human infection with H6 viruses in 2013 [51], as well as detection of H6 antibodies [12,14] indicate that H6 viruses can cross species barriers and infect humans.

Our results showed that H6N2 AIVs circulating in Mediterranean/Black Sea and West Asian/East African flyways have several molecular markers which may be associated with elevated zoonotic potential. All four H6N2 viruses harbored 29-to-31 molecular markers that may provide selective advantage for the viruses in mammalian hosts in terms of pathogenicity, increased polymerase activity, mammalian adaptation and/or antiviral resistance (Figure 1, Table 1, Table S3). Most of the markers were identified in the polymerase genes (15-to-16 markers) (Table 1). These markers may increase the stability of the polymerase complex to function efficiently in mammalian hosts, hence may provide adaptation to mammals. One such example was located in the PB2 gene of EG/1566C, S590/R591, which was also present in 2009 pandemic H1N1 influenza viruses [26]. For mammalian adaptation, increased affinity for α-2,6 SA receptors is also important. We detected one such marker in HA, A156 (H5 numbering), which may provide adaptation to mammals due to increased affinity of HA towards α-2,6 SA receptor [40,41]. In addition to the markers that function individually, we also detected markers that function when they coexist. One such example is the presence of V89, D309, K339, G477, V495, T676 together in the PB2 of all four H6N2 viruses that increases the replication and virulence in mammals [25]. These six markers are found in the PB2 genes of 1957 H2N2 and 2009 H1N1 pandemic viruses, as well. Five of these markers (except T676) are also found in the PB2 of 1968 H3N2 pandemic virus. Furthermore, we detected markers in NP (V105) and NS1 (A149) that may provide adaptation to poultry which may also increase the possibility for human encounter with the virus. Considering the coexistence of almost ~30 amino acid markers in their genome, which may confer adaptive advantage in both mammals and poultry, the H6N2 isolates of Turkey, Egypt, and Uganda may have potential for zoonosis. However, taking into consideration that the effect of molecular markers on the pathogenicity, transmissibility or antiviral resistance in mammals could be subtype-specific, further investigation is required to evaluate the zoonotic potential of avian origin H6N2 viruses by conducting in vitro and in vivo assays.

Turkey has always been an important but missing piece of the global AIV puzzle, connecting Europe and Asia. In addition, there is a clear gap in AIV surveillance in Sub-Saharan Africa. Since the viruses in this study were isolated around the same time and genetically quite closer to each other, there is a possibility that the virus was carried to different locations via comigrating birds. Only the virus isolated in Uganda was rather distant from the other viruses, but it still showed high nucleotide and amino acid similarities with the remaining three viruses. This variance might be due to the fact that the Ugandan virus was isolated approximately six to seven months before (Summer 2017) the Turkish and Egyptian viruses (Winter 2018); thus, it may represent a different migration season. Reassortment events have been characterized here but the origins of each gene segment are difficult to assess because of the surveillance gap in the region. Thus, our data underlines the importance of regular surveillance studies in the region, and across the Mediterranean/Black Sea and West Asian/East African flyways to obtain more comprehensive and complete understanding on AIV evolution.

## Figures and Tables

**Table 1:** Number of amino acid markers of mammalian adaptation, pathogenicity, and antiviral resistance identified in each protein of H6N2 viruses.

Protein	GD/18	UG/731	EG/1566C	EG/1556OP
**PB2**	7	7	8	7
**PB1**	7	7	7	7
**PB1-F2**	2	1	1	1
**PA**	4	4	4	4
**PA-X**	2	2	2	2
**HA**	1	2	2	2
**NP**	1	0	1	1
**NA**	1	1	1	1
**M1**	2	2	2	2
**M2**	0	0	0	0
**NS1**	3	3	3	3
**NEP**	0	0	0	0
**TOTAL**	30	29	31	30

**Table 2:** Genetic constellations of Ugandan, Egyptian, and Turkish isolates.

Protein	Uganda	Egypt	Turkey
**PB2**	A/domestic duck/Georgia/1/2016 (H4N6)-like	A/Teal/Markeev/13-3-11/2016 (H5N2) and A/teal/Egypt/MB-D-487OP/2016(H7N3)-like	
**PB1**	A/teal/Egypt/MB-D-487OP/2016 (H7N3) and A/duck/Moscow/ 5662/2018 (H1N2)-like	A/domestic duck/Georgia/1/2016 (H4N6)-like	
**PA**	A/teal/Egypt/MB-D-487OP/2016 (H7N3)-like	A/Teal/Markeev/13-3-11/2016 (H5N2)-like	
**HA**	A/domestic duck/Georgia/1/2015(H6N2)-like		
**NP**	A/teal/Egypt/MB-D-487OP/2016 (H7N3) and A/domestic duck/Georgia/1/2016 (H4N6)-like		A/Teal/Markeev/13-3-11/2016 (H5N2)-like
**NA**	A/duck/Moscow/5662/2018 (H1N2)-like	A/Teal/Markeev/13-3-11/2016 (H5N2)-like	
**M**	A/Teal/Markeev/13-3-11/2016 (H5N2), A/domestic duck/Georgia/1/2016 (H4N6), A/duck/Moscow/5662/2018 (H1N2), and A/teal/Egypt/MB-D-487OP/2016 (H7N3)-like		
**NS**	A/domestic duck/Georgia/1/2016 (H4N6) and A/teal/Egypt/MB-D-487OP/2016 (H7N3)-like		

Note: The Georgian and Egyptian reference viruses (A/domestic duck/Georgia/1/2016 (H4N6) and A/teal/Egypt/MB-D-487OP/2016 (H7N3)) originated from locations at the overlap between the Mediterranean/Black Sea and the West Asian/East African flyways. The Ukrainian and Russian viruses (A/Teal/Markeev/13-3-11/2016 (H5N2) and A/duck/Moscow/5662/2018 (H1N2)) originated from the Mediterranean/Black Sea flyway.

## Data Availability

The sequence data of all four isolates are deposited into GenBank and are accessible with the following accession numbers: MT783429 to MT783436 for GD/18; MT783429 to MT783436 for UG/731; MT570358 to MT570365 for EG/1566C and MT570350 to MT570357 for EG/1556OP.

## Supplementary Materials

The following are available online at https://www.mdpi.com/article/10.3390/v13040607/s1, Table S1. Pairwise nucleotide similarities of virus isolates. Table S2. Pairwise amino acid similarities of virus isolates. Table S3. Amino acid markers reported in the literature to be associated with host tropism, increased virulence, and escape from the host immune system. Figure S1. Phylogenetic relationships between GD/18, EG/1566C, EG/1556OP and UG/731 based on PB1 genes. Figure S2. Phylogenetic relationships between GD/18, EG/1566C, EG/1556OP and UG/731 based on PA genes. Figure S3. Phylogenetic relationships between GD/18, EG/1566C, EG/1556OP and UG/731 based on NP genes. Figure S4. Phylogenetic relationships between GD/18, EG/1566C, EG/1556OP and UG/731 based on M genes. Figure S5. Phylogenetic relationship between GD/18, EG/1566C, EG/1556OP and UG/731 based on NS gene.
