# Supplementary material for: Molecular Characterization of Closely Related H6N2 Avian Influenza Viruses Isolated from Turkey, Egypt, and Uganda
Source: Viruses. 2021 Apr 2;13(4):607. doi: 10.3390/v13040607 (PMC8065897; doi:10.3390/v13040607)
Supplement: Supplementary file 1 [file viruses-13-00607-s001.pdf]

Molecular Characterization of Closely Related H6N2 Avian Influenza Viruses Isolated from Turkey, Egypt, and Uganda

Yavuz Mercan [1,2,†], Gladys Atim [3,†], Ahmed E. Kayed [4,†], M. Ekin Azbazdar [1,2], Ahmed Kandeil [4], Mohamed A. Ali [4], Adam Rubrum [5], Pamela McKenzie [5], Richard J. Webby [5], Bernard Erima [3], Fred Wabwire-Mangen [3,6], Qouilazoni A. Ukuli [3], Titus Tugume [3], Denis K. Byarugaba [3,7], Ghazi Kayali [8,9], Mariette F. Ducatez [10], and Zeynep A. Koçer [1,2,*]

1 Emerging Viral Diseases Laboratory, Izmir Biomedicine and Genome Center, Izmir, 35340 Turkey; yavuz.mercan@msfr.ibg.edu.tr (Y.M.); ekin.azbazdar@msfr.ibg.edu.tr (M.E.A.)
2 Izmir International Biomedicine and Genome Institute, Dokuz Eylul University, Izmir, 35340 Turkey
3 Makerere University Walter Reed Project, P.O. Box 7062 Kampala, Uganda gatim@muwrp.org (G.A.); berima@muwrp.org (B.E.); fwabwire@musph.ac.ug (F.W.-M.); qukuli@muwrp.org (Q.A.U.); ttugume@muwrp.org (T.T.); dkb@covab.mak.ac.ug (D.K.B.)
4 Center of Scientific Excellence for Influenza Viruses, National Research Centre, Giza, 12311 Egypt; Ahmed.Elsayed@human-link.org (A.E.K.); Ahmed.Kandeil@human-link.org (A.K.); mohamedahmedali2004@yahoo.com (M.A.A.)
5 St Jude Children's Research Hospital, Memphis, TN, 38105 USA; Adam.Rubrum@stjude.org (A.R.); Pamela.McKenzie@stjude.org (P.M.); richard.webby@stjude.org (R.J.W.)
6 School of Public Health, Makerere University, P.O. Box 7062 Kampala, Uganda
7 College of Veterinary Medicine, Makerere University, P.O. Box 7062 Kampala, Uganda
8 Department of Epidemiology, Human Genetics, and Environmental Sciences, University of Texas, Houston, TX, 77030 USA; ghazi@human-link.org (G.K.)
9 Human Link, Dubai, United Arab Emirates
10 IHAP, UMR1225, Université de Toulouse, INRAe, ENVT, 31076 Toulouse, France; mariette.ducatez@envt.fr (M.F.D.)
† These authors contributed equally to this work
* Correspondence: zeynep.kocer@ibg.edu.tr (Z.A.K.); Tel.: +90-232-299-4165

Citation: Lastname, F.; Lastname, F.; Lastname, F. Title. *Viruses* **2021**, *13*, x. https://doi.org/10.3390/xxxxx

Academic Editor: Firstname Lastname

Academic Editor: Firstname Lastname

Received: 27 February 2021
Accepted: 30 March 2021
Published: date

Publisher's Note: MDPI stays neutral with regard to jurisdictional claims in published maps and institutional affiliations.

Copyright: by the authors. Licensee MDPI, Basel, Switzerland. This article is an open access article distributed under the terms and conditions of the Creative Commons Attribution (CC BY) license (http://creativecommons.org/licenses/by/4.0/).

Table S1. Pairwise nucleotide similarities of virus isolates.

Gene		GD/18	UG/731	EG/1566C	EG/1556OP
**PB2**	GD/18	1			
	UG/731	0.91506	1		
	EG/1566C	0.9853	0.91082	1	
	EG/1556OP	0.9978	0.91564	0.98484	1
**PB1**	GD/18	1			
	UG/731	0.961213	1		
	EG/1566C	0.97354	0.96076	1	
	EG/1556OP	0.999824	0.960759	0.99912	1
**PA**	GD/18	1			
	UG/731	0.951806	1		
	EG/1566C	0.997671	0.950274	1	
	EG/1556OP	0.998138	0.950787	0.99535	1
**HA**	GD/18	1			
	UG/731	0.966591	1		
	EG/1566C	0.982039	0.966632	1	
	EG/1556OP	0.981418	0.965985	0.999411	1
**NP**	GD/18	1			
	UG/731	0.949747	1		
	EG/1566C	0.953384	0.975428	1	
	EG/1556OP	0.953384	0.975428	1	1
**NA**	GD/18	1			
	UG/731	0.947689	1		
	EG/1566C	0.998772	0.948232	1	
	EG/1556OP	0.998772	0.948232	1	1
**M**	GD/18	1			
	UG/731	0.975291	1		
	EG/1566C	0.9967	0.976016	1	
	EG/1556OP	0.9967	0.9760161	1	1
**NS**	GD/18	1			
	UG/731	0.984703	1		
	EG/1566C	0.979922	0.971398	1	
	EG/1556OP	0.979922	0.971398	1	1

Table S2. Pairwise amino acid similarities of virus isolates.

Protein		GD/18	UG/731	EG/1566C	EG/1556OP
**PB2**	GD/18	1			
	UG/731	0.994785	1		
	EG/1566C	0.994785	0.989542	1	
	EG/1556OP	1	0.994785	0.994785	1
**PB1**	GD/18	1			
	UG/731	0.996029	1		
	EG/1566C	0.998678	0.994702	1	
	EG/1556OP	1	0.996029	0.998678	1
**PB1-F2**	GD/18	1			
	UG/731	0.966098	1		
	EG/1566C	0.988827	0.977527	1	
	EG/1556OP	0.988827	0.977527	1	1
**PA**	GD/18	1			
	UG/731	0.992991	1		
	EG/1566C	0.998602	0.994398	1	
	EG/1556OP	0.998602	0.994398	1	1
**PA-X**	GD/18	1			
	UG/731	0.984	1		
	EG/1566C	0.996024	0.979959	1	
	EG/1556OP	0.996024	0.979959	1	1
**HA**	GD/18	1			
	UG/731	0.97314	1		
	EG/1566C	0.99113	0.98217	1	
	EG/1556OP	0.98934	0.98037	0.99823	1
**NP**	GD/18	1			
	UG/731	0.989991	1		
	EG/1566C	0.987879	0.993958	1	
	EG/1556OP	0.987879	0.993958	1	1
**NA**	GD/18	1			
	UG/731	0.95227	1		
	EG/1566C	0.99575	0.95707	1	
	EG/1556OP	0.99575	0.95707	0.99165	1
**M1**	GD/18	1			
	UG/731	1	1		
	EG/1566C	1	1	1	
	EG/1556OP	1	1	1	1
**M2**	GD/18	1			
	UG/731	0.989637	1		
	EG/1566C	1	0.989637	1	
	EG/1556OP	1	0.989637	1	1
**NS1**	GD/18	1			
	UG/731	0.968955	1		
	EG/1566C	0.995624	0.964598	1	
	EG/1556OP	0.995624	0.964598	1	1
**NEP**	GD/18	1			
	UG/731	0.991701	1		
	EG/1566C	0.991701	0.983333	1	
	EG/1556OP	0.991701	0.983333	1	1

Table S3. Amino acid markers reported in the literature to be associated with host tropism, increased virulence, and escape from the host immune system.

Protein	Amino Acid Marker	Phenotype	Viruses with the marker	Reference(s)[c]
**PB2**	I63 (with PB1 T677)	Pathogenic in mice	All	[27]
	V89 (with, D309, K339, G477, V495, T676)	Enhanced polymerase activity, Increased virulence in mice	All	[25]
	S590/R591	Increased polymerase activity, Human host adaptation	EG/1566C	[26]
**PB1**	V3 (with N328 and N375)	Increased polymerase activity, Increased virulence in ferrets and mice	All	[28, 31]
	P13	Increased polymerase activity, Adaptation to mice	All	[30]
	Y436	Increased polymerase activity and virulence in mallards, ferrets and mice	All	[29]
	V473	Increased polymerase activity and replication efficiency in mammalian cells	All	[32]
	T677 (with PB2 I63)	Pathogenic in mice	All	[27]
**PB1-F2**	S66	Increased virulence in mammals	All	[34, 35]
	L82	Increased pathogenicity in mice	GD/18	[36]
**PA**	A37	Significantly increased viral growth and polymerase activity in mammalian cells	All	[38]
	S277 (with Q278 and P653)	Adaptation to mammalian hosts	All	[37]
**PA-X**	P28	Increased host shutdown in mammalian cells	All	[39]
	S65	Increased host shutdown in mammalian cells	All	[39]
**HA**[a]	A156	Increased virus binding to α2–6 sialic acid receptor, Transmissibility in Guinea pigs	All	[40, 41]
	T189	Increased virus binding to α2–6 SA receptor	UG/731, EG/1566C, EG/1556OP	[42]
**NP**	V105	Adaptation of duck AIVs to chickens	GD/18, EG/1566C, EG/1556OP	[43]
**NA**[b]	T117	Reduced susceptibility to oseltamivir and zanamivir	All	[44]
**M1**	D30	Increased virulence in mice	All	[45]
	A215	Increased virulence in mice	All	[45]

**NS1**	S42	Increased virulence in mice, Antagonism of IFN induction	All	[47]
	F138 (with ESEV in PDZ binding domain)	Increased replication in mammalian cells, Decreased interferon response	All	[46]
	A149	Increased virulence and decreased interferon response in chickens	All	[48]

No amino acid substitution was detected from the literature for M2 nor NEP (NS2) proteins.

[a]According to H5 numbering

[b]According to N2 numbering

[c]Reference numbers were given in accordance with the references in the main text

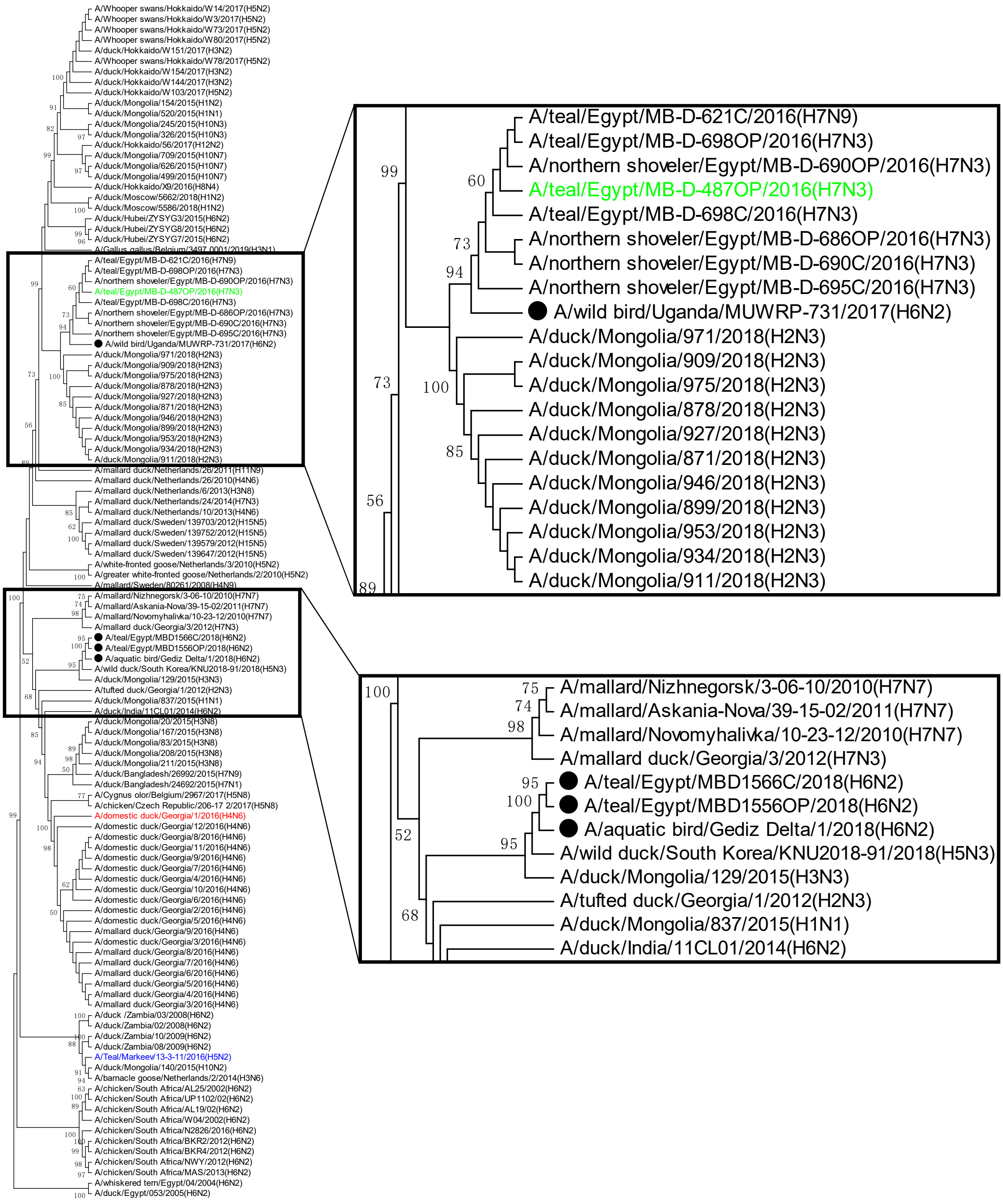

Figure S1. Phylogenetic relationships between GD/18, EG/1566C, EG/1556OP and UG/731 based on PB1 genes. All four viruses were indicated with black circles. Phylogenetic trees were constructed using the Maximum Likelihood method based on the Hasegawa-Kishino-Yano model. PB1 genes of GD/18, EG/1566C and EG/1556OP clustered with Korean and Mongolian sequences. PB1 gene of UG/731 mainly located in the same cluster with Egypt and Mongolia PB1 sequences. Reference viruses (most closely related strains for at least one gene segment) are color-coded: A/domestic duck/Georgia/1/2016 (H4N6) in red, A/Teal/Markeev/13-3-11/2016 (H5N2) in blue, and A/teal/Egypt/MB-D-487OP/2016 (H7N3) in green.

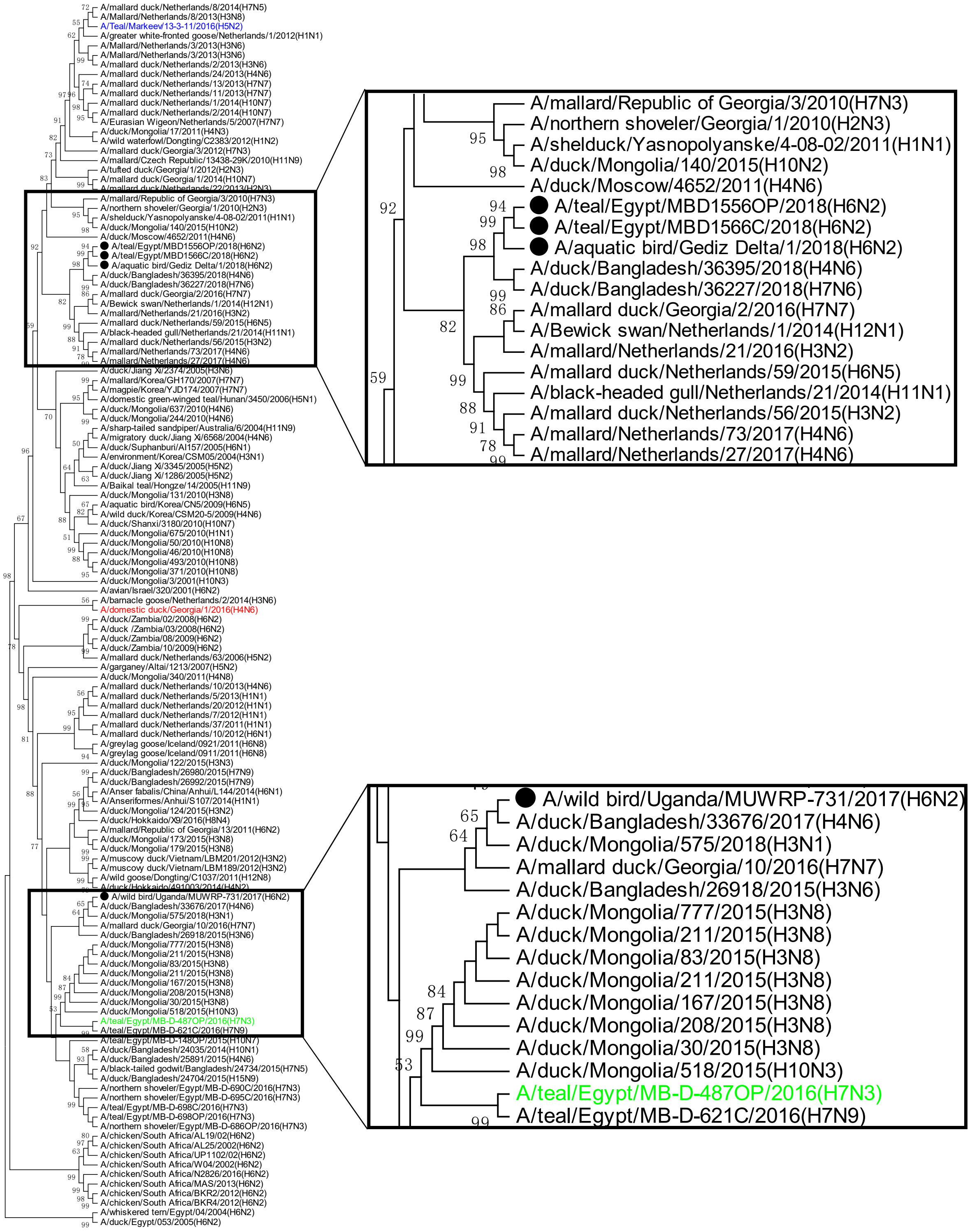

Figure S2. Phylogenetic relationships between GD/18, EG/1566C, EG/1556OP and UG/731 based on PA genes. All four viruses were indicated with black circles. Phylogenetic trees were constructed using the Maximum Likelihood method based on the Hasegawa-Kishino-Yano model. PA genes of GD/18, EG/1566C and EG/1556OP clustered together with Bangladesh and Netherlands sequences. PA of UG/731 clustered with Mongolia, Egypt, and Bangladesh sequences. Reference viruses (most closely related strains for at least one gene segment) are color-coded: A/domestic duck/Georgia/1/2016 (H4N6) in red, A/Teal/Markeev/13-3-11/2016 (H5N2) in blue, and A/teal/Egypt/MB-D-487OP/2016 (H7N3) in green.

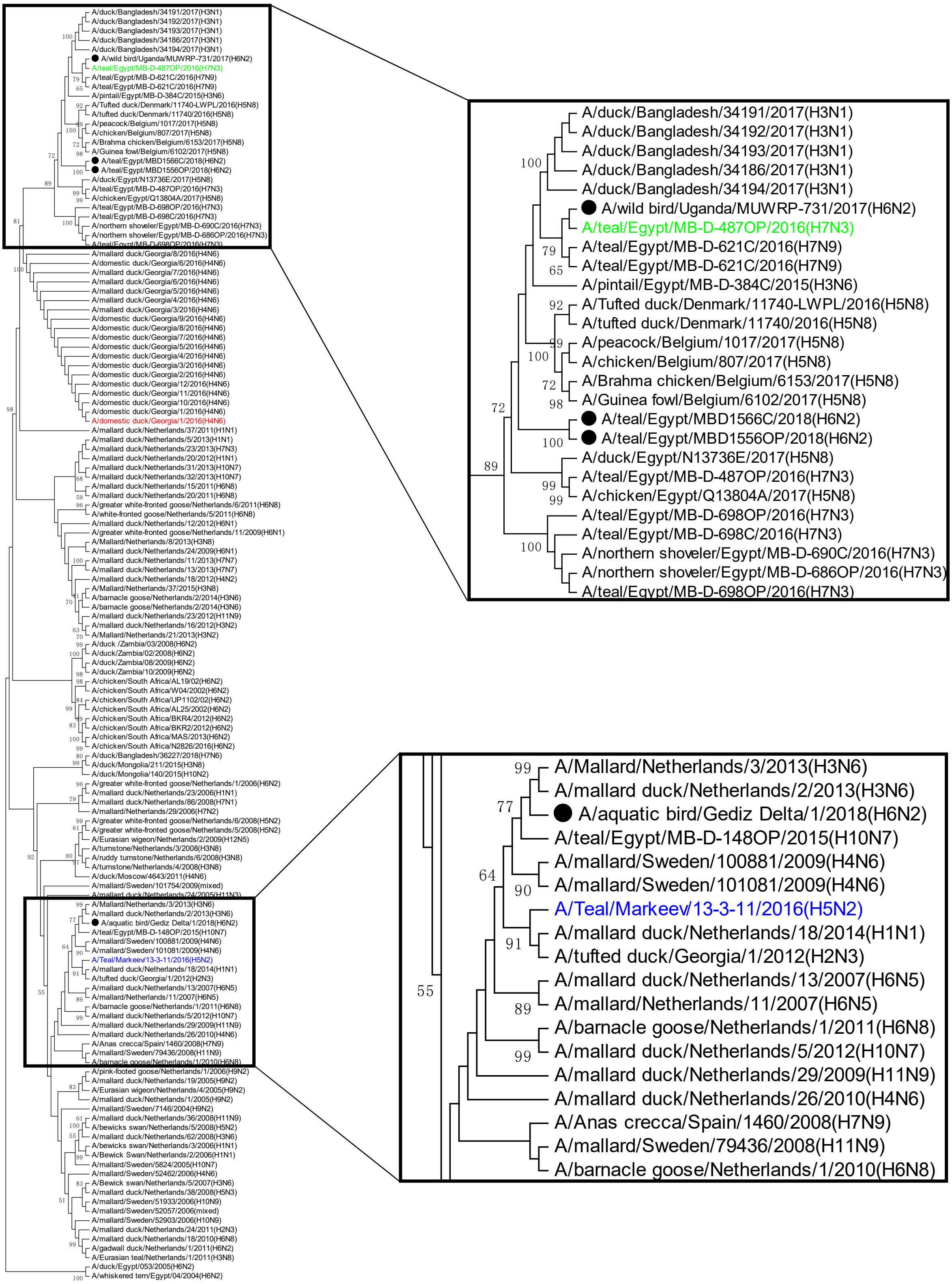

Figure S3. Phylogenetic relationships between GD/18, EG/1566C, EG/1556OP and UG/731 based on NP genes. All four viruses were indicated with black circles. Phylogenetic trees were constructed using the Maximum Likelihood method based on the Hasegawa-Kishino-Yano model. NP of UG/731, EG/1566C and EG/1556OP clustered with Bangladesh, Egypt, and some European sequences. NP of GD/18 clustered with European sequences. Reference viruses (most closely related strains for at least one gene segment) are color-coded: A/domestic duck/Georgia/1/2016 (H4N6) in red, A/Teal/Markeev/13-3-11/2016 (H5N2) in blue, and A/teal/Egypt/MB-D-487OP/2016 (H7N3) in green.

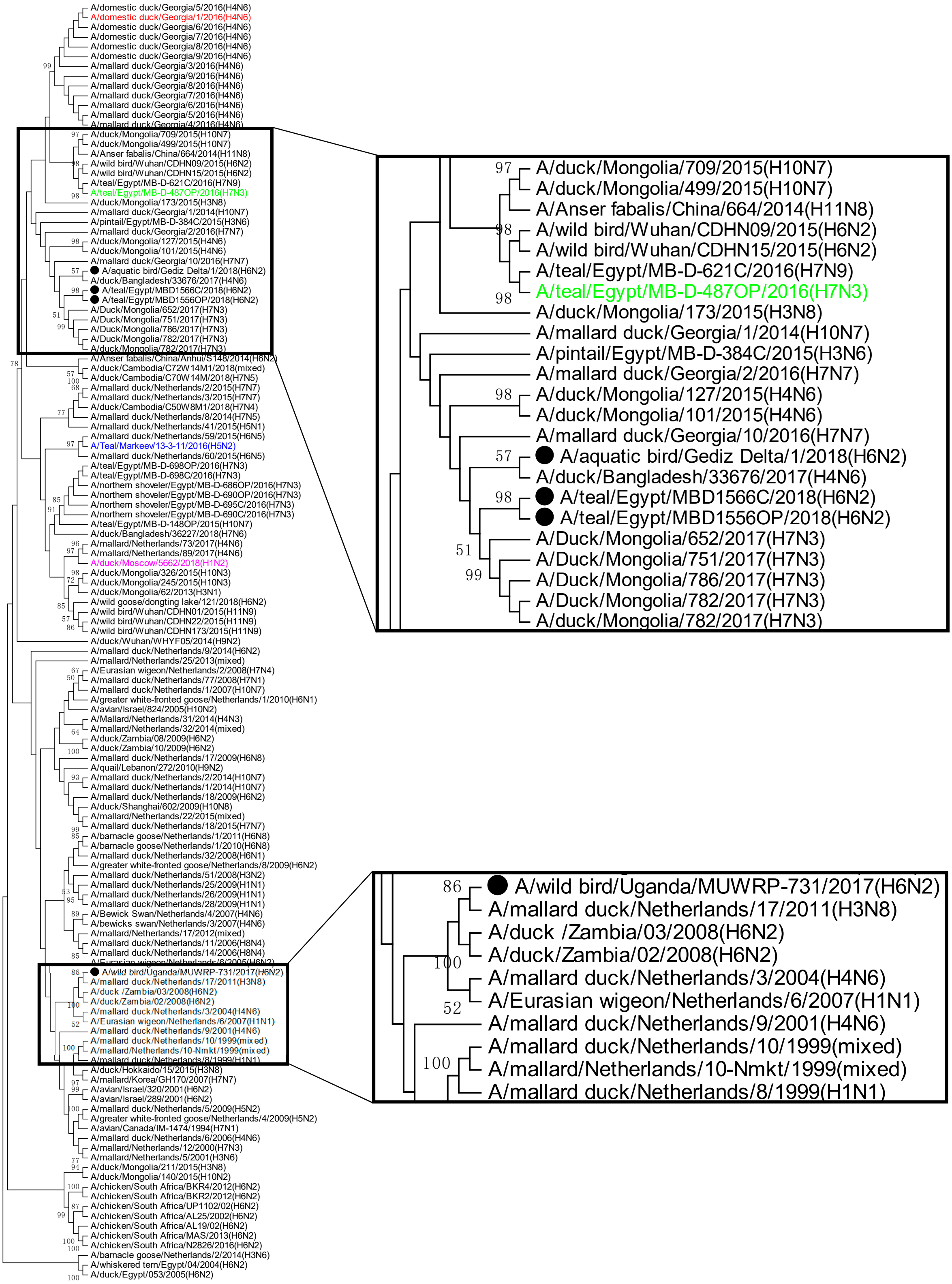

Figure S4. Phylogenetic relationships between GD/18, EG/1566C, EG/1556OP and UG/731 based on M genes. All four viruses were indicated with black circles. Phylogenetic trees were constructed using the Maximum Likelihood method based on the Hasegawa-Kishino-Yano model. M gene sequences of GD/18, EG/1566C and EG/1556OP clustered together with Bangladesh, Mongolia, and Georgia sequences. M gene of UG/731 clustered with Netherlands sequences. Reference viruses (most closely related strains for at least one gene segment) are color-coded: A/domestic duck/Georgia/1/2016 (H4N6) in red, A/Teal/Markeev/13-3-11/2016 (H5N2) in blue, A/teal/Egypt/MB-D-487OP/2016 (H7N3) in green, and A/duck/Moscow/5662/2018 (H1N2) in purple.

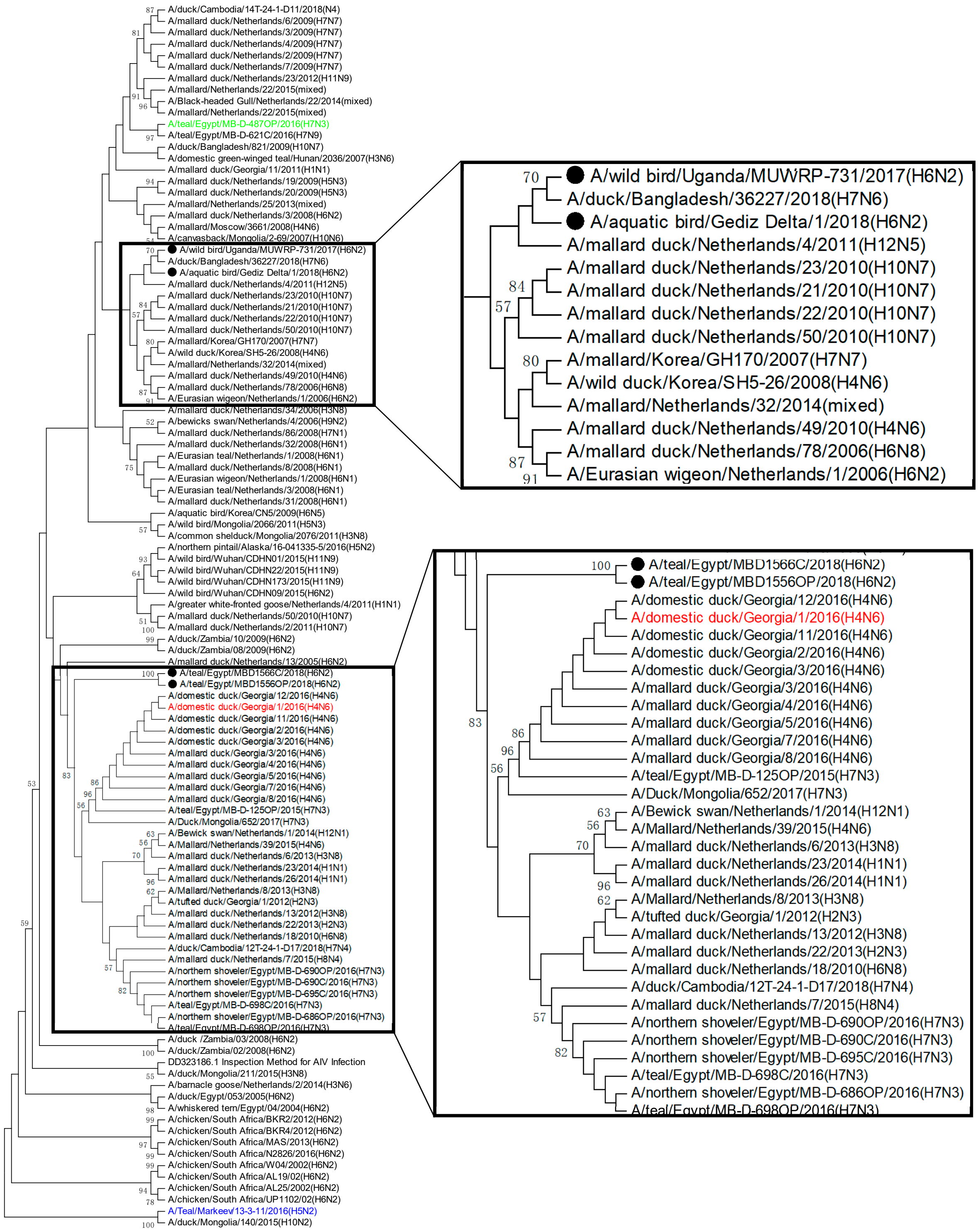

Figure S5. Phylogenetic relationship between GD/18, EG/1566C, EG/1556OP and UG/731 based on NS gene. All four viruses were indicated with black circles. Phylogenetic tree was constructed using the Maximum Likelihood method based on the Hasegawa-Kishino-Yano model. NS gene of EG/1566C and EG/1556OP clustered together Georgia, Netherlands, and Egypt sequences. NS of UG/731 and GD/18 clustered with Bangladesh, Korea, and Netherland sequences. Reference viruses (most closely related strains for at least one gene segment) are color-coded: A/domestic duck/Georgia/1/2016 (H4N6) in red, A/Teal/Markeev/13-3-11/2016 (H5N2) in blue, and A/teal/Egypt/MB-D-487OP/2016 (H7N3) in green.
